# Gene panel screening for insight towards breast cancer susceptibility in different ethnicities

**DOI:** 10.1371/journal.pone.0238295

**Published:** 2020-08-31

**Authors:** Madison R. Bishop, Sophonie M. Omeler-Fenaud, Anna L. W. Huskey, Nancy D. Merner

**Affiliations:** 1 Harrison School of Pharmacy, Department of Drug Discovery and Development, Auburn University, Auburn, Alabama, United States of America; 2 College of Veterinary Medicine, Department of Pathobiology, Auburn University, Auburn, Alabama, United States of America; CNR, ITALY

## Abstract

African American breast cancer genetics is less understood compared to European American breast cancer susceptibility. Despite the many advantages of gene panel screening, studies investigating African American inherited breast cancer risk and comparing variant contributions between ethnicities are infrequent. Thus, 97 breast cancer-affected individuals of African and European descent from the Alabama Hereditary Cancer Cohort were screened using the research-based gene-panel, B.O.P. (**B**reast, **O**varian, and **P**rostate cancer). Upon sequencing and bioinformatic processing, rare coding variants in 14 cancer susceptibility genes were categorized according to the American College of Medical Genetics guidelines and compared between ethnicities. Overall, 107 different variants were identified, the majority of which were benign/likely benign. A pathogenic/likely pathogenic variant was detected in 8.6% and 6.5% of African American and European American cases, respectively, which was not statistically significant. However, African Americans were more likely to have at least one variant of uncertain significance (VUS; *p-value* 0.006); they also had significantly more VUSs in *BRCA1/2* compared to European Americans (*p-value* 0.015). Additionally, 51.4% of African Americans and 32.3% of European Americans harbored multiple rare variants, and African Americans were more likely to have at least one VUS and one benign/likely benign variant (*p-value* 0.032), as well as multiple benign/likely benign variants (*p-value* 0.089). Moreover, of the 15 variants detected in multiple breast cancer cases, *ATM* c.2289T>C (p.F763L), a VUS, along with two likely benign variants, *BRCA2* c.2926_2927delinsAT (p.S976I) and *RAD51D* c.251T>A (p.L84H), were determined to be associated with African American breast cancer risk when compared to ethnic-specific controls. Ultimately, B.O.P. screening provides essential insight towards the variant contributions in clinically relevant cancer susceptibility genes and differences between ethnicities, stressing the need for future research to elucidate inherited breast cancer risk.

## Introduction

The introduction of next-generation sequencing (NGS) and implementation of gene-panel screening have significantly reduced sequencing costs and has enabled the analysis of multiple genes-of-interest simultaneously [1, 2]. As such, gene panels can be used in both clinical or research settings to identify variants in genes known to harbor variants that cause or increase the risk of disease [3, 4]. Despite these advantages, few efforts have been published on gene-panel testing in minority populations, especially regarding the assessment of hereditary breast cancer risk. Breast cancer gene-panel screenings have focused on cohorts of mostly white, non-Hispanic individuals [2]; only a small number of studies sought to exclusively examine populations of Asian [5–7] or African [8, 9] descent. However, a few screening reports of multi-ethnic breast cancer cohorts have been published, which have compared variant contributions between ethnicities [10–13]. Even with these efforts, African American breast cancer genetics is less understood compared to the genetic susceptibility of individuals of European descent [8].

There are many breast cancer disparities between African and European Americans [14]. Despite European Americans having higher incidence rates between the ages of 65–84, African American women have higher rates before the age of 40. Knowing that early age of onset is a hallmark of hereditary breast cancer [1], genetic risk factors may be contributing to this disparity. Interestingly, African Americans have been reported to have more variants of uncertain significance (VUSs) in clinically valid breast/ovarian cancer genes [10–13, 15, 16], which warrant further investigation using NGS gene panels. Ultimately, a complete spectrum of breast cancer risk variants needs to be defined to provide greater insight towards African American breast cancer disparities.

The Alabama Hereditary Cancer Cohort (AHCC) provides an opportunity to study breast cancer genetics in underrepresented individuals [17]. Alabama is a medically underserved state and 26.8% of its population self-identify as being Black or African Americans, which is double the national percentage. African American breast cancer probands, which are seemingly unrelated cases, represent 37% of the AHCC due to the focused effort to include this minority population [17]. Thus, 97 breast cancer-affected individuals of either African or European descent from the AHCC were screened using the research-based gene-panel, B.O.P. (**B**reast, **O**varian, and **P**rostate cancer) [4, 17]. Rare variants in 14 cancer susceptibility genes were assessed and compared between ethnicities.

## Materials and methods

Auburn University Institutional Review Board approved this study. Study participants were recruited and enrolled into a cancer genetic study through IRB-approved protocols, 14–232, 14–335, and 15–111. Informed consent was obtained in writing from all study participants. In total, 97 (35 African American and 62 European American) seemingly unrelated breast cancer cases were selected from the AHCC for genetic analysis based on sequential enrollment. The specific recruitment and enrollment efforts involved for the AHCC were previously described [17]. With average ages of onsets of 45.7 and 47.4 years for African Americans and European Americans, respectively, this cohort represented breast cancer-affected individuals who enrolled into the study because of a young age at diagnosis (<45 years of age) and/or a family history of the disease, which are characteristics of hereditary breast cancer [17]. Genomic DNA from each individual was screened using the custom-designed gene panel, B.O.P., which targets genes that are either clinically proven or thought to be associated with risk of breast, ovarian and/or prostate cancer [4]. DNA libraries were prepared following the HaloPlex HS Target Enrichment System For Illumina Sequencing Protocol (Version C0, December 2015) and subsequently sequenced on an Illumina HiSeq^™^ 2500 at the Genomic Services Laboratory at HudsonAlpha Institute for Biotechnology. Following capture and sequencing, variants were called using an in-house bioinformatics pipeline [4].

Fourteen genes that were targeted on the B.O.P. panel and have National Comprehensive Cancer Network (NCCN) clinical management guidelines regarding the genetic risk of breast cancer and/or ovarian cancer [18] were selected for variant analysis: *ATM* (NM_000051), *BARD1* (NM_000465), *BRCA*1 (NM_007300), *BRCA2* (NM_000059), *CDH1* (NM_004360), *BRIP1* (NM_032043), *CHEK2* (NM_001005735), *NBN* (NM_002485), *PALB2* (NM_024675), *PTEN* (NM_000314), *RAD51C* (NM_058216), *RAD51D* (NM_001142571), *STK11* (NM_000455), *TP53* (NM_000546). The depth of coverage of each assessed gene was calculated using DepthOfCoverage tool within the GATK (v.3.4–46) and ranged from 408X-970X (S1 Table). Only variants within coding regions of the 14 genes were further investigated. Next, variants were filtered using ethnic-specific minor allele frequency (MAF) of ≤1% from controls in the National Heart, Lung, and Blood Institute (NHLBI) Exome Sequencing Project Exome Variant Server (EVS) [19]. The EVS data is publicly available and was downloaded as a merged VCF file for each assessed gene. Additionally, known sequencing artifacts from previous screening and validation were removed [4].

After filtering, true positives were identified according to criteria established through B.O.P.’s initial analytical assessment [4]. As a result, true positives included variants with high confidence calls (depth of coverage ≥100X and alternate allele frequency ≥40%), as well as variants with low confidence calls (depth of coverage <100X and alternate allele frequency <40%) that were subsequently validated through polymerase chain reactions (PCR) and Sanger sequencing. All true positive variants were organized into American College of Medical Genetics (ACMG) variant categories for clinical interpretation (pathogenic, likely pathogenic, variant of uncertain significance (VUS), benign, and likely benign) according to InterVar [20, 21]. As recommended, due to InterVar’s automated interpretation based on default parameters, some variant classifications were manually adjusted. Specifically, classifications corresponding to the most recent entries in ClinVar from reputable companies (i.e. Ambry Genetics, Invitae, GeneDx) were considered [22]. Using the Fisher’s exact test in R (v 3.5.1), the number of breast cancer-affected individuals with a particular variant, as well as variants in different categories and genes were compared between ethnicities. Furthermore, of the variants identified in more than one breast cancer-affected individual of the same ethnicity, allele frequencies were compared between cases and ethnic-specific controls from EVS [19].

## Results

**Fig 1:**
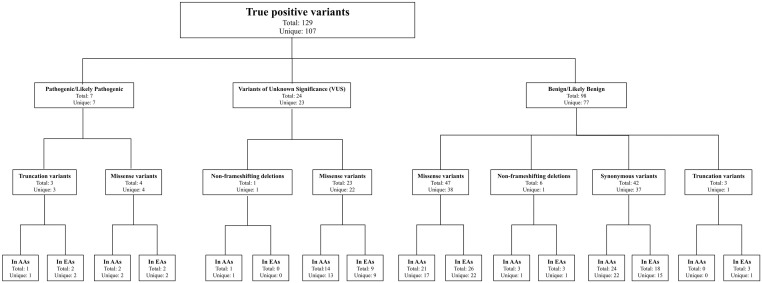
ACMG classifications of variants detected after B.O.P. gene panel screening, bioinformatics processing, and filtering. (AAs) African Americans; (EAs) European Americans.

**Fig 2:**
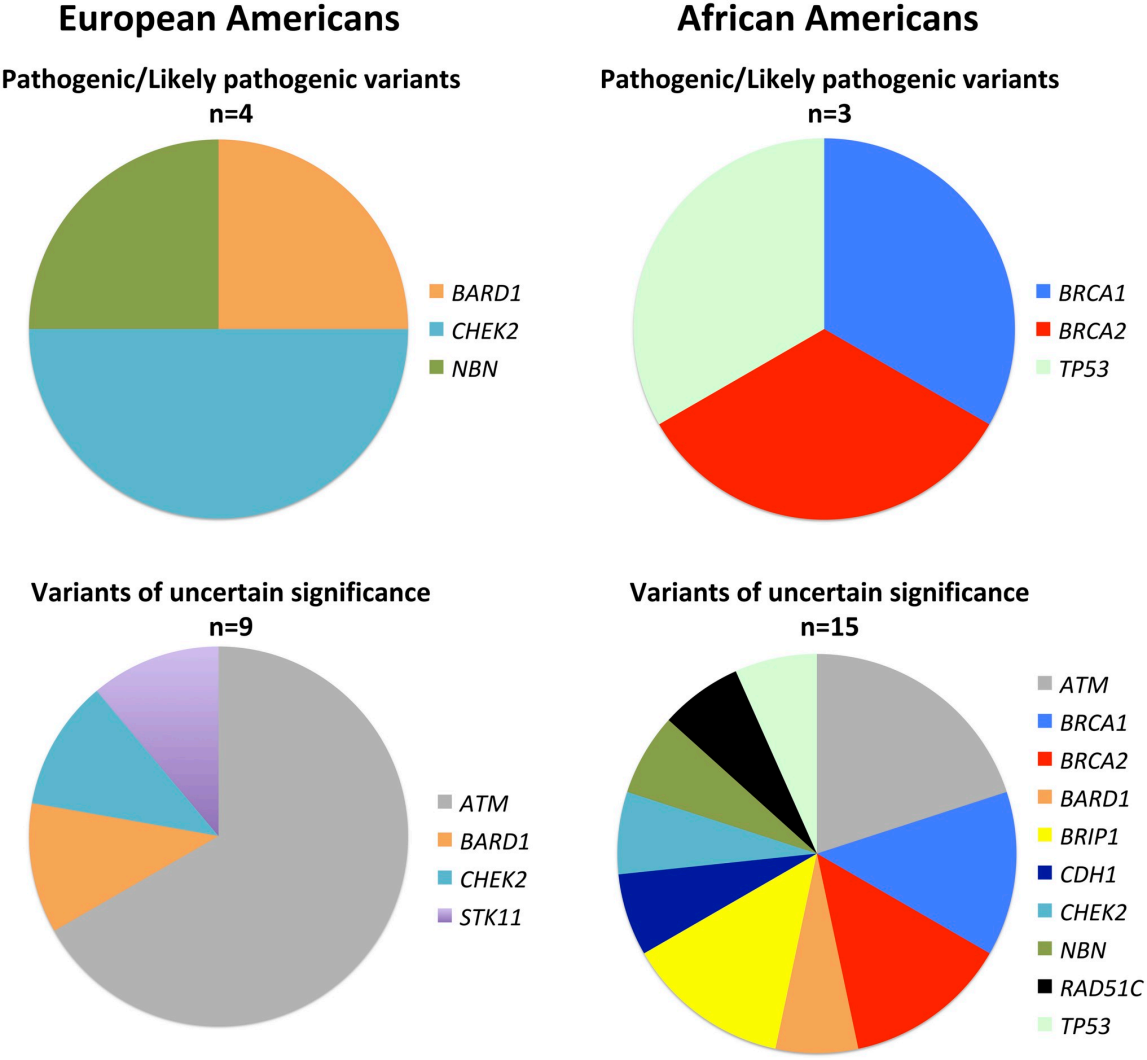
Genes harboring pathogenic/likely pathogenic variants and VUS.

Overall, 107 different (unique), rare coding variants were classified as true positives; since 15 of the unique variants were found in multiple individuals, a total of 129 true positives were identified (Fig 1 and S2 Table). Most variants were benign/likely benign; however, a total of seven pathogenic/likely pathogenic variants were identified as well as 24 VUSs (Figs 1 and 2). The seven different pathogenic/likely pathogenic variants were each detected in one individual, three African Americans and four European Americans (Table 1). The gene distributions were vastly different for each ethnicity (Fig 2). Overall, a pathogenic/likely pathogenic variant was detected in 7.2% (7/97) of all the screened breast cancer cases, corresponding to 8.6% of African American cases and 6.5% of European American cases, which is not a statistically significant difference (Table 2).

**Table 1:** Pathogenic/Likely pathogenic variants detected after B.O.P. screening 97 breast cancer affected cases.

Gene Name	Chr	Position (hg38)	Ref. Allele	Alt. Allele	Exon	DNA Change	Amino Acid Change	Protein Function	ACMG classification	Number of BC cases with variant	
										EA	AA
*BARD1*	chr2	214728861_214728862	TG	-	exon 11	c.2148_2149del	p.T716fs	Truncation	Pathogenic	1	0
*BRCA1*	chr17	43051071	A	C	exon 21	c.5387T>G	p.M1796R	Missense	Pathogenic	0	1
*BRCA2*	chr13	32339966_ 32339970	AGTAA	-	exon 11	c.5611_5615del	p.S1871fs	Truncation	Pathogenic	0	1
*CHEK2*	chr22	28725099	A	G	exon 5	c.599T>C	p.I200T	Missense	Pathogenic	1	0
		28725338	T	C	exon 4	c.478A>G	p.R160G	Missense	Likely Pathogenic	1	0
*NBN*	chr8	89982770	G	-	exon 2	c.123delC	p.I41fs	Truncation	Pathogenic	1	0
*TP53*	chr17	7673776	G	A	exon 8	c.844C>T	p.R282W	Missense	Pathogenic	0	1

(AA) African American; (ACMG) American College of Medical Genetics; (Alt.) Alternate; (BC) Breast cancer; (Chr) Chromosome; (EA) European American; (Ref.) Reference.

**Table 2:** Ethnic comparisons between different variant categories.

Variant categories	AA BC cases		EA BC cases		Comparison of AA and EA BC cases
	# of cases	Percentage	# of cases	Percentage	p-values*
at least one variant of any category	31	88.6%	40	64.5%	0.016
at least one pathogenic/likely pathogenic variant	3	8.6%	4	6.5%	0.700
at least one VUS	14	40.0%	9	14.5%	0.006
at least one benign/likely benign variant	25	71.4%	36	58.1%	0.274
multiple variants of any category	18	51.4%	20	32.3%	0.084
multiple pathogenic/likely pathogenic variants	0	0.0%	0	0.0%	1
multiple VUS	1	2.9%	0	0.0%	0.361
multiple benign/likely benign variants	13	37.1%	12	19.4%	0.089
at least one pathogenic variant and one VUS	1	2.9%	1	1.6%	1
at least one pathogenic variant and one benign/likely benign variant	1	2.9%	3	4.8%	1
at least one VUS and one benign/likely benign variant	9	25.7%	5	8.1%	0.032

(AA) African American; (BC) Breast cancer; (EA) European American;

*p-values generated using Fisher’s Exact Test.

African American breast cancer cases were more likely to harbor at least one rare variant in one of the 14 assessed susceptibility genes compared to European American breast cancer cases (*p-value* 0.016; Table 2). This finding was highly influenced by the ethnic differences in VUSs. African Americans were more likely to have at least one VUS (*p-value* 0.006; Table 2 and Fig 2); they also had significantly more VUSs in *BRCA1/2* compared to European Americans (*p-value* 0.015; Table 3). Additionally, 51.4% of African American breast cancer cases and 32.3% of European American breast cancer cases harbored multiple rare variants amidst the 14 genes (Table 2). African Americans were more likely to have at least one VUS and one benign/likely benign variant (*p-value* 0.032); African Americans also had more breast cancer cases with multiple benign/likely benign variants, resulting in a *p-value* trending toward significance (*p-value* 0.089; Table 2).

**Table 3:** Ethnic comparisons between VUS in different BC susceptibility genes.

Genes with VUS	AA BC cases with VUS		EA BC cases with VUS		Comparison of AA and EA BC cases
	# of cases	Percentage	# of cases	Percentage	p-values*
*BRCA1/2*	4	11.4%	0	0.0%	0.015
*ATM*	3	8.6%	6	9.7%	1
*BARD1*	1	2.9%	1	1.6%	1
*BRIP1*	2	5.7%	0	0.0%	0.128
*CDH1*	1	2.9%	0	0.0%	0.361
*CHEK2*	1	2.9%	1	1.6%	1
*NBN*	1	2.9%	0	0.0%	0.361
*RAD51C*	1	2.9%	0	0.0%	0.361
*STK11*	0	0.0%	1	1.6%	1
*TP53*	1	2.9%	0	0.0%	0.361

(AA) African American; (BC) Breast cancer; (EA) European American;

*p-values generated using Fisher’s Exact Test.

As mentioned above, there were 15 variants detected in multiple breast cancer cases; this included five and seven variants detected solely in African American and European American breast cancer cases, respectively (S3 Table). All of those variants were classified as benign/likely benign except *ATM* c.2289T>C (p.F763L), which is a VUS. Interestingly, *ATM* c.2289T>C (p.F763L), along with two other variants currently classified as likely benign, *BRCA2* c.2926_2927delinsAT (p.S976I) and *RAD51D* c.251T>A (p.L84H), were determined to be associated with African American breast cancer risk when compared to ethnic-specific controls (Table 4). Furthermore, comparing all 15 variants between African and European American breast cancer cases, *BRCA2* c.2926_2927delinsAT (p.S976I), which was solely detected in African American cases, was the only variant statistically more likely to be observed in either ethnic group (*p-value* 0.044; Table 4 and S3 Table).

**Table 4:** Variants identified in more than one breast cancer-affected individual of the same ethnicity and associated with breast cancer risk.

Gene Name	Chr	Start position (hg38)	Ref. Allele	Alt. Allele	Exon	DNA Change	Amino Acid Change	Protein Function	ACMG Classification	Number of BC cases with variant		Comparison between EA and AA BC cases	Population control comparison (EVS)			
										EA	AA	*p-value*	AA MAF (%)	AA Alt. allele count	AA WT allele count	AA BC risk p-value*
*ATM*	chr11	108257519	T	A	exon 15	c.2289T>A	p.F763L	Missense	VUS	0	2	0.128	0.14	6	4396	0.006
*BRCA2*	chr13	32337281_32337282	TC	AT	exon 11	c.2926_2927delinsAT	p.S976I***	Missense	Likely benign	0	3	0.044	0	0	4406	3.67 x 10^−6^
*RAD51D*	chr17	35116931	A	T	exon 3	c.251T>A	p.L84H	Missense	Likely benign	0	2	0.128	0.26	8	3128	0.019

(AA) African American; (ACMG) American College of Medical Genetics; (Alt.) Alternate; (BC) Breast cancer; (Chr) Chromosome; (EA) European American; (EVS) Exome Variant Server; (Ref.) Reference;

*p-values generated using Fisher’s Exact Test.

## Discussion

Involving underrepresented individuals in cancer genetics research is crucial to better understand inherited risk in different ethnicities. Herein, 97 breast cancer-affected individuals from the AHCC [17] were screened using the B.O.P. gene panel [4] to identify rare variants (MAF ≤1%) in 14 cancer susceptibility genes and compare the spectrum of variants between African and European Americans. The 14 assessed genes are clinically valid; the NCCN has established breast and/or ovarian cancer risk management guidelines regarding genetic testing results for each of the genes [18]. The variants identified during this study were categorized according to ACMG guidelines, which were established for clinical interpretation [21].

A pathogenic/likely pathogenic variant was detected in seven breast cancer cases, representing 7.2% of the total cohort and corresponding to 8.6% and 6.5% of African American and European American cases, respectively. This slightly higher frequency in African Americans was not statistically significant but was similarly observed in a recent report by Jones *et al*. [13]. Though it is typically reported that closer to 20% of hereditary breast cancer cases have a high-risk, pathogenic variant in a clinically relevant gene [1], the percentage of cases in this study with such variants is lower. However, it is worth noting that the 14 assessed genes only represent a fraction of the susceptibility genes listed in the NCCN breast/ovarian cancer genetic screening guidelines [18]. For instance, the Lynch syndrome genes *MLH1*, *MSH2*, *MSH6*, *PMS2*, and *EPCAM* are included in the guidelines to be managed based on family history [18] and are commonly screened during breast cancer genetic risk assessment [23, 24], but were not included in this analysis. Furthermore, variants in other cancer susceptibility genes (i.e., *MUTYH* and *CDKN2A*) are sometimes reported through breast cancer risk assessment [10], but were not assessed in this study. Ultimately, the assessment of this particular number and group of genes may explain the lower percentage of detected pathogenic/likely pathogenic variants. It may also simply be explained by the unique patient population. Ultimately, similar to typical breast cancer gene screening efforts, these results emphasize that the majority of African and European American individuals with familial/hereditary breast cancer remain genetically unsolved upon gene screening [1, 3, 8]; thus, pursuing discovery efforts is important.

*BRCA1/2* are recognized as the most commonly mutated genes in hereditary breast cancer cases [1]. Yet, there were no *BRCA1/2* pathogenic/likely pathogenic variants detected in European American cases in this study. This finding is contrary to the results of African Americans for which *BRCA1/2* variants represented 67% of the detected pathogenic/likely pathogenic variants. Even though it was unexpected to not observe any *BRCA1/2* variants in European Americans, our results corroborate a recent study by Kurian *et al*. that reported more *BRCA1* pathogenic variants in African American compared to European American breast cancer cases [12]. Kurian *et al*. also reported that *CHEK2* pathogenic variants were more common in European Americans compared to other minorities. We observed similar results, detecting pathogenic/likely pathogenic *CHEK2* variants solely in European Americans. Overall, with African Americans reported to have high genetic diversity and a unique spectrum of variants [25], it is not surprising that no pathogenic/likely pathogenic variants overlapped between ethnicities. In fact, each pathogenic/likely pathogenic variant was unique to an individual.

Previously, in our initial publication describing the B.O.P. panel, we assessed coding variants (in a subset of clinically valid breast/ovarian cancer genes) with MAFs ≤2% in cancer-affected cases from the AHCC and identified a significant difference in the number of African Americans with at least one variant compared to European Americans (*p-value* 2.71 X 10^−3^) [4]. Similarly, this study revealed that significantly more African American breast cancer cases had at least one rare variant (MAF ≤1%) in the 14 assessed genes compared to European American breast cancer cases (89% versus 65%, respectively; *p-value* 0.016). This occurrence was primarily a result of the difference in VUSs, being in 40% and 14.5% of African Americans and European Americans, respectively (*p-value* 6.45 X 10^−3^). Even though VUSs were identified in 11 of the 14 assessed genes, there were significantly more *BRCA1/2* VUSs in African Americans compared to European Americans. Such differences have been reported since some of the earliest multi-ethnic *BRCA1/2* screening studies [15, 16] and continue to be reiterated in multi-ethnic gene panel studies [10, 12, 13]. Thus, in conjunction with those studies, our findings not only reinforce that more inclusive research studies need to be carried out but emphasize the need to investigate this class of variants further. Functional studies, family segregation analyses, and large association studies are pertinent for determining the actual pathogenicity of each identified VUS, which will ultimately result in variant reclassification [3, 26, 27]. This is essential because clinical management does not change with the identification of a VUS [18], and African Americans are disparately receiving such inconclusive results.

Even though ACMG guidelines have been developed for the clinical interpretation of genetic variants in clinically valid susceptibility genes [21], in reality, classification still varies amongst different clinical laboratories, and variant reclassification is an issue [27, 28]. In addition to VUSs, which most frequently undergo reclassifications, the clinical impact of variants in other categories can be downgraded or upgraded [27]. Even though over ~90% of variant reclassifications are downgrades and less than 10% of reclassifications result in a change of actionability, it has been demonstrated that of variants that undergo a change in actionability, 64% are upgrades and 34% are downgrades [27]. Thus, in other words, the majority of variant reclassifications that result in a change in actionability reclassify benign/likely benign variants or VUSs to pathogenic/likely pathogenic. In saying that, it is important to note that there were three variants in this study that were associated with African American breast cancer risk, *ATM* c.2289T>C (p.F763L), which is a VUS, and two other variants currently classified as likely benign, *BRCA2* c.2926_2927delinsAT (p.S976I) and *RAD51D* c.251T>A (p.L84H). Considering that reclassification rates vary by ancestry and are highest in ethnic minorities [29], these variants could eventually undergo an upgrade in clinical impact; thus, further investigation is warranted. However, similar to *BRCA2* c.9976A>T; p.K3326X, they may be low-risk variants, which are currently not clinically relevant [18, 30, 31]. *BRCA2* c.9976A>T; p.K3326X, which we identified in three European American breast cancer cases, is classified as likely benign according to the ACMG guidelines. Ultimately, to truly understand risk, all risk variants will need to be considered, no matter where they fall on the spectrum.

NGS, including gene panel screening, detects the full spectrum of variants in the targeted region(s) for each individual screened, which provides an opportunity to explore how combinations of variants contribute towards polygenic risk [32]. Although recent efforts examining polygenic risk of breast cancer have focused on common variants [31], rare variants that modify risk in *BRCA1* and *BRCA2* mutation carriers have been identified [33, 34]. Considering this, assessing combinations of rare variants is likely a vital missing component for polygenic breast cancer risk assessment. In our study, 51.4% of African American breast cancer cases and 32.3% of European American cases had multiple rare variants in the 14 clinically relevant cancer susceptibility genes (*p-value* 0.084). This overall difference seemed to be specifically related to more African Americans having multiple benign/likely benign variants (*p-value* 0.089), as well as at least one VUS and one benign/likely benign variant (*p-value* 0.032), the latter being statistically significant. Despite that some of the VUSs could eventually be re-classified as pathogenic/likely pathogenic and associated with high risk, overall these variants may individually only slightly elevate risk and specific combinations of these variants may multiplicatively influence risk of developing breast cancer. Therefore, comparing such rare variant combinations between cases and ethnic-specific controls using NGS approaches will provide essential insight towards polygenic breast cancer risk, particularly in African Americans [3]. This effort requires having individual sequencing files from each assessed control, which were not available for this study.

Lastly, with the launch of NGS, several whole-exome sequencing investigations have been carried out to identify novel breast cancer risk variants; however, the majority of those studies were relatively unsuccessful due to the heterogeneity of breast cancer genetics [1]. Noteworthy, the successful whole-exome sequencing studies focused on relatively homogeneous populations [1, 35], suggesting that investigating homogeneous cohorts is a useful approach to enhance our understanding of breast cancer genetics. By screening cancer cases from the AHCC, which was established through strategic recruitment mechanisms that involved traveling to isolated and rural communities in Alabama, the detection of ancestral mutations in seemingly unrelated individuals was anticipated [17]. Overall, this B.O.P. screening suggests that the AHCC is relatively homogeneous since a total of 15 rare variants in the 14 cancer susceptibility genes were detected in multiple seemingly unrelated breast cancer cases. This occurrence likely facilitated the African American breast cancer associations regarding *ATM* c.2289T>C (p.F763L), *BRCA2* c.2926_2927delinsAT (p.S976I) and *RAD51D* c.251T>A (p.L84H). Additionally, while this study focused on variants with ethnic-specific MAF≤1%, a previous B.O.P. analysis identified a slightly more common, synonymous variant (*STK11* c.369G>A;p.Q123Q) associated with African American breast cancer (*p-value* 8.50 X 10^−4^) when compared to ethnic-specific controls (MAF of 1.5%) [4]. Nonetheless, the publicly available controls used in this study are not the ideal comparison, being a compilation of cohorts that were sequenced on a different NGS platform [19], and screening larger cohorts including both affected cases as well as internal controls is required to validate these preliminary findings, considering the small sample size in this study. Overall, this study provides insight towards the variant contributions in clinically relevant cancer susceptibility genes and the differences between European and African Americans. Future research should broaden the search for potential genetic risk factors to include all variant types and combinations. Expanding the scope will elucidate breast cancer genetics and potentially identify the hereditary factors that play a role in the disparate number of early-onset breast cancers observed in African American women.

## Supporting information

S1 Table Summary of coverage for the fourteen assessed genes from the B.O.P. panel. (XLSX)

S2 Table All true positive variants. (XLSX)

S3 Table Variants detected in multiple breast cancer cases. (AA) African American; (Alt.) Alternate; (BC) Breast cancer; (Chr) Chromosome; (EA) European American; (#) esp6500siv2; (Het) Heterozygous; (MAF) minor allele frequency; (Ref.) Reference; (VUS) Variant of Uncertain Significance.
(XLSX)
